# The Children and Young People’s Books Lexicon (CYP-LEX): A large-scale lexical database of books read by children and young people in the United Kingdom

**DOI:** 10.1177/17470218241229694

**Published:** 2024-03-12

**Authors:** Maria Korochkina, Marco Marelli, Marc Brysbaert, Kathleen Rastle

**Affiliations:** 1 Department of Psychology, Royal Holloway, University of London, Egham, UK; 2 Department of Psychology, University of Milano-Bicocca, Milan, Italy; 3 Department of Experimental Psychology, Ghent University, Ghent, Belgium

**Keywords:** Lexical database, children’s books, reading, lexical statistics, word frequency

## Abstract

This article introduces the Children and Young People’s Books-Lexicon (CYP-LEX), a large-scale lexical database derived from books popular with children and young people in the United Kingdom. CYP-LEX includes 1,200 books evenly distributed across three age bands (7–9, 10–12, 13+) and comprises over 70 million tokens and over 105,000 types. For each word in each age band, we provide its raw and Zipf-transformed frequencies, all parts-of-speech in which it occurs with raw frequency and lemma for each occurrence, and measures of count-based contextual diversity. Together and individually, the three CYP-LEX age bands contain substantially more words than any other publicly available database of books for primary and secondary school children. Most of these words are very low in frequency, and a substantial proportion of the words in each age band do not occur on British television. Although the three age bands share some very frequent words, they differ substantially regarding words that occur less frequently, and this pattern also holds at the level of individual books. Initial analyses of CYP-LEX illustrate why independent reading constitutes a challenge for children and young people, and they also underscore the importance of reading widely for the development of reading expertise. Overall, CYP-LEX provides unprecedented information into the nature of vocabulary in books that British children aged 7+ read, and is a highly valuable resource for those studying reading and language development.

## Introduction

Learning to read is one of the most important milestones of a child’s schooling. The ability to decode written symbols and understand their meaning is a vehicle for learning and forms the basis for an individual’s social and cultural development, professional success, and overall prosperity. Substantial research has focused on the importance of high-quality systematic phonics instruction in the initial years of learning to read (e.g., Ehri et al., 2001). However, becoming a skilled reader is a process that arises over many years and that requires more than classroom instruction (see Castles et al., 2018, for a comprehensive review). In particular, the transition from novice to expert reader requires substantial text experience (e.g., Mol & Bus, 2011). This experience is thought to allow emerging readers the opportunity to encounter different words repeatedly (e.g., Castles et al., 2007; Share, 1999; Zevin & Seidenberg, 2002), and in different contexts (e.g., Nation, 2017), thereby building higher-quality lexical representations that free cognitive resources for comprehension (e.g., Perfetti & Hart, 2002).

The building of lexical quality (and therefore reading fluency) is reliant on children engaging in independent reading (Share, 2004). Yet, we know very little about what children and young people are reading and the nature of words that they encounter. To address this gap, we built a large-scale lexical database, the Children and Young People’s Books Lexicon (CYP-LEX), based on books for children aged 7–16 that are popular in the United Kingdom. In the remainder of this section, we provide a brief overview of the existing children’s corpora in English, including the main findings from these corpora, and demonstrate why the databases derived from these corpora are unsatisfactory for research questions concerning reading acquisition. We then move on to describe how the CYP-LEX database was constructed and the information that is available in this database. Finally, we report what CYP-LEX reveals about book vocabulary, in particular, how book vocabulary compares to vocabulary on British television, and how it changes as readers transition into and through adolescence.

### Existing corpora of children’s books

To date, three lexical databases of children’s books used for independent reading in English^1^ have been developed: the Corpus-based Learning about Language in the Primary-school (Thompson & Sealey, 2007), the Children’s Printed Word Database (Masterson et al., 2010), and the Oxford Children’s Corpus (Wild et al., 2013).^2^

The *Corpus-based Learning about Language in the Primary-school* (CLLIP) was compiled from a subset of 30 imaginative fiction texts for primary school children (8–10 years) from the British National Corpus (BNC) and contains 698,286 tokens (number of types, i.e., unique words, was not reported). Thompson and Sealey (2007) compared CLLIP with two adult corpora, a corpus of adult imaginative fiction (*N* texts = 317; 12,869,883 tokens) and a corpus of newspaper texts (*N* texts = 114; 1,270,798 tokens), both extracted from the BNC. All three corpora were found to be very similar in terms of the most frequent words; however, unlike the two fiction corpora, the newspaper corpus had no personal pronouns among its 10 most common words. The children and adult fiction corpora contained similarly high proportions of nouns (around 15%–17% of all types), lexical verbs (around 14%), and adverbs (around 8%). In contrast, the newspaper corpus was reported to have many more nouns (23% of all types) and fewer lexical verbs (around 10%) than the two fiction corpora. These observations are interesting and provide valuable insights into the children’s texts lexicon, however, because the CLLIP corpus was derived from a very small selection of homogeneous texts, Thompson and Sealey’s (2007) conclusions are best treated with caution. The CLLIP corpus was never made publicly available.

The *Children’s Printed Word Database* (CPWD) originally contained frequencies for 12,193 words (derived from 995,927 tokens) extracted from 1,011 books for children aged 5–9 years (Masterson et al., 2010). This database was designed to serve as an extension of another, much smaller, word list based on 685 books read by children in Year 1 of a school in North London (Stuart et al., 2003). In the case of CPWD, book selection relied on the results of a school survey, in which teachers selected from around the United Kingdom indicated which of the books included in popular reading schemes such as Ginn 360 or Oxford Reading Tree were read by children in their classes (Reception through Year 3). Masterson et al. (2010) reported a marked increase in lexical diversity between Reception and the following years. However, they also concluded that the CPWD vocabulary was dramatically skewed towards the lower frequencies: half of the types were used so rarely that they accounted for only about 2.1% of all tokens. In contrast, the top 100 words (just under 1% of all types) were so high in frequency that they comprised half of all tokens. Unfortunately, the CPWD is no longer available. Moreover, since the survey that determined inclusion of books into CPWD was conducted in 2003, it is contentious to what extent the CPWD vocabulary is representative of what British primary school child-ren are reading today.

Finally, the *Oxford Children’s Corpus* (OCC) is a lexical database derived from books for children aged from 5 to 14 and was designed to represent a wide range of educational stages (or Key Stages, as defined in the UK National Curriculum), genres, and time periods. When introducing the OCC, Wild et al. (2013) reported that it contained approximately 31 million tokens, whereby 1.8 million tokens were graded as Key Stage 1 (5–7 years), 14 million tokens were classified as Key Stage 2 (7–11 years), and 8 million tokens as Key Stage 3 (11–14 years). Three-quarters of the OCC (approx. 23 million tokens) were derived from fiction texts for children, while the rest consists of nonfiction texts (about 7 million tokens) and samples of writing by children (approx. 1.4 million tokens). Half of the tokens in the OCC were obtained from texts written in the 21st century, while the other half were extracted from texts that originated in the 20th century (approx. 12 million tokens) or earlier (4 million tokens). Importantly, while one of the major advantages of a corpus such as the OCC is that it is being updated regularly and should therefore reflect the most recent developments in children’s literature (as reflected in reading materials published by Oxford University Press), it has restricted access and is primarily used for commercial purposes and solely in projects approved by Oxford University Press. The word lists (i.e., types) derived from the OCC have never been made publicly available.

Summing up, two of the previously developed corpora of children’s books in English are small, target narrow age cohorts (CLLIP: 8–10 years, CPWD: 5–9 years), and are likely to be outdated as they were both constructed more than 20 years ago. Most importantly, none of the three corpora and the resulting databases is publicly available and can be used freely for research purposes. The CYP-LEX database, being publicly available and comprising 1,200 books that are popular among British children and young people today, was intended to fill this gap.

### Previous investigations of vocabulary in children’s books

In literate societies, children are exposed to book reading from an early age. Children’s early experience with print has a major impact on the development of literary skills (e.g., Lonigan et al., 2008; Puranik et al., 2011), and, once children have learned to read, most new words that they learn are likely to be acquired through reading (Nagy & Herman, 1984). The most prominent line of research on children’s book vocabulary has focused on how language in books targeted at pre-readers (e.g., picture books) differs from language observed in child–parent interactions. The main aim of this research has been to understand whether and how shared book reading can facilitate reading acquisition. In parallel, a smaller literature attempted to characterise the vocabulary used in books designed for independent reading during primary and secondary schooling, whereby the focus has been on understanding how specific properties of written language (e.g., morphological complexity) change as book target age increases. In the following paragraphs, we provide a brief overview of the main findings from these two literatures.

There is strong scientific consensus that language in picture books is much richer than the spoken language that pre-readers experience. Montag et al. (2015) compared picture book vocabulary with vocabulary used in spontaneous verbal interactions between young children and their parents, playmates, and teachers. The corpus of picture books was derived from a pool of 100 picture books recommended for shared reading with preschoolers under the age of 6, and samples of child-directed speech were extracted from the American English subcorpus of the freely available Child Language Data Exchange System (CHILDES) database (MacWhinney, 2000, 2001). Montag et al. (2015) observed that picture books had greater lexical density (the ratio of the number of unique words to the overall text size) and diversity (proportion of content words in a text sample), suggesting greater breadth of vocabulary and denser information content than in child-parent conversational speech. Recently, Dawson et al. (2021) replicated these findings for the British English subcorpus of CHILDES, and they also showed that picture books had greater lexical sophistication (i.e., higher proportion of rare words) than day-to-day conversations. Dawson and colleagues also reported that picture book vocabulary contained more adjectives and nouns than child-directed speech, and that these words were longer, and more abstract and emotionally arousing than those used in speech, while they also contained more affixes and had a later age of acquisition. Finally, Massaro (2015) reported that children were about 3 times more likely to encounter a new low-frequency word while listening to a reading of a picture book relative to listening to their care-giver’s speech. These findings have been taken to suggest that exposure to text via read-aloud experiences may bene-fit vocabulary acquisition and possibly reading comprehension once children begin to learn to read.

Relative to the study of picture book vocabulary, there is much less research on vocabulary in books written for independent reading in English, primarily due to a lack of large publicly available corpora of texts that children and adolescents read (see previous section). We have reported above that two of the existing corpora of children’s books were derived from small samples of largely homogeneous reading materials aimed at younger primary school child-ren and that these corpora are not publicly available. The largest-to-date corpus of children’s books is thus the OCC (also not publicly available). Recently, Dawson et al. (2023) used a subset of OCC texts for children between 5 and 14 years (Key Stages 1–3) to study how the morphological complexity (as indexed by the number of affixed words) of these texts changed as a function of text target age and genre. While nonfiction texts were found to contain a higher proportion of complex words overall (i.e., higher proportion of complex word tokens), fiction texts were reported to include more unique multimorphemic words (i.e., higher proportion of complex word types). Dawson et al. (2023) further showed that the number of morphologically complex words (tokens and types) increased in line with book target age, and that this increase was attributable to an increase in the number of tokens for derived (e.g., “teacher”), but not compound (e.g., “football”) or compound-derived (e.g., “footballer”) words. In the pool of texts examined in this study, multimorphemic words occurred less frequently and were rated as more abstract and later acquired than their monomorphemic counterparts. This initial investigation of morphological complexity provides valuable insights into how book vocabulary changes as reading experience builds; however, due to a small corpus size (e.g., 14 nonfiction texts for Key Stage 1), further work is required to ascertain whether Dawson et al.’s (2023) findings are replicable with a larger corpus.

Summing up, there is ample evidence that language that children encounter in (English) picture books through shared reading departs substantially from language in child–parent or adult interactions. We are not aware of any large-scale investigations of language in books aimed at older readers; to date, only one study has examined voca-bulary in books for children and adolescents, focusing specifically on differences in morphological complexity in books aimed at children of different ages (Dawson et al., 2023). The novel large-scale lexical database introduced in this article provides an opportunity to systematically describe various aspects of vocabulary in books that child-ren read. In the following sections, we describe how the texts contained in the CYP-LEX corpus were collected and pre-processed, what information is available in the resulting database, and some initial insights that this database reveals about children’s books vocabulary.

## Method

### Corpus collection

In building the CYP-LEX database, our goal was to create a resource that would allow researchers to study how vocabulary in the books that children read changes as child-ren grow older. The reading experience of a 7-year-old is likely to differ from that of a 14-year-old in many different ways, and we wished to create a database that would capture these differences. Therefore, our approach was to build a corpus in which there was an equal number of books in each age band rather than a corpus in which the age bands have an equal number of tokens. Older children can read (and enjoy) books that are likely to pose an insurmountable challenge for most of their younger counterparts, for reasons to do with both book length (i.e., the number of tokens) and overall language complexity. Thus, building equally-sized age bands would have resulted in equating the reading experience of children of different ages and reading abilities, a scenario which is far from reality.

The CYP-LEX corpus thus comprises 1,200 books, evenly distributed across three age bands, 400 books for 7- to 9-year-olds, 400 books for 10- to 12-year-olds, and 400 books for children aged 13+. The books were selected based on book popularity statistics from national reading surveys (e.g., Topping et al., 2022), British reading charities (e.g., BookTrust, National Literacy Trust), book cataloguing websites (e.g., UK book lists on Goodreads, Common Sense Media, The School Reading List, LoveReading4Kids, Read Brightly), British book retailers (e.g., Waterstones), British publishers (e.g., Penguin), and book sales statistics and recommendations from Amazon UK. The vast majority of books in the CYP-LEX corpus were included in most of the sources listed above, suggesting a high level of agreement regarding the books that are most popular. For the 13+ age band, we also included books (N=32) that were part of the General Certificate of Secondary Education (GCSE) assessment in English li-terature, since virtually every pupil in England reads a selection of these books. Perhaps unsurprisingly, most books in the above-mentioned lists were fiction; however, each age band also includes a small percentage of nonfiction books (5%, 9%, and 6.5% for 7–9, 10–12, and 13+ age bands, respectively). The list of books included in the corpus is provided in Supplementary material A (https://doi.org/10.17605/OSF.IO/SQU49); however, due to copyright restrictions, we cannot make the full textual content of the books available.

### Text cleaning and pre-processing

Once selected, the books were either purchased as e-books or, in the case of older books for which the copyright has expired, downloaded for free from Project Gutenberg, and then converted to text using custom-made Python code. As part of the conversion procedure, the books were stripped from all metadata (e.g., books’ front and back matter, except for glossaries and timelines) and manually checked for major conversion errors (e.g., missing spaces, unrecognised symbols). By the end of the pre-processing pipeline, each book was stored in the form of a .txt file, in which the text was lowercase, quotation marks were removed, contractions expanded, and individual sentences separated by line-breaks.

The .txt files were then submitted to the Stanford CoreNLP language processing tool (Manning et al., 2014) that takes in raw text and runs a series of natural language processing (NLP) annotators.^3^ We used the Stanford CoreNLP server through Python with the following annotators: “tokenize” (tokenises text), “ssplit” (splits text into sentences), “pos” (adds part-of-speech tags), “lemma” (associates each token with a lemma, i.e., the unmarked form of a set of inflected word forms), and “ner” (adds named-entity tags). For part-of-speech tagging, Stanford CoreNLP uses the Penn Treebank tag set (see Toutanova et al., 2003, for algorithm implementation, and Marcus et al., 1993, for a detailed documentation of the tag set), and, for lemmatisation, it relies on the algorithm proposed by Minnen et al. (2001), which has been estimated to achieve 97% accuracy (Brysbaert et al., 2012).

Following annotation, the corpus, containing 71,296,246 tokens and 323,670 types, was cross-re-ferenced against the Spell Checker Oriented Word Lists (SCOWL) database (http://wordlist.aspell.net/). This database consists of over 658,235 English words, including extremely rare words, abbreviations, acronyms, spelling variants in different English dialects, and proper names. Tokens that were not present in the SCOWL database were deemed invalid and removed after ensuring that the vast majority of these resulted from character recognition errors.

## The CYP-LEX database

**Figure 1.**
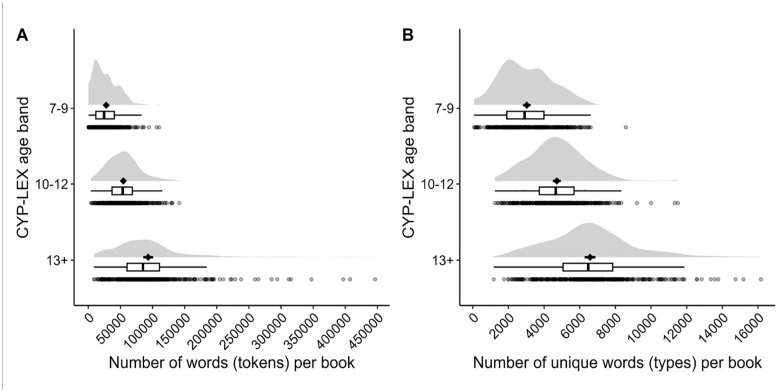
Distribution of tokens (panel A) and types (panel B) across the books and age bands. For each age band, the graph shows the density plot (the “cloud”) with the mean (the black diamonds) and two standard errors of the mean (the black error bars), the number of tokens and types in each book within that age band (the dots), and the interquartile range (the boxes signify the upper and the lower quartiles, and the horizontal lines represent the data outside the upper and lower quartiles), with vertical black lines representing the median.

The resulting corpus comprises 70,287,217 tokens, and the database, containing 105,694 types, can be accessed on this project’s OSF website (https://doi.org/10.17605/OSF.IO/SQU49). Table 1 reports the number of tokens, types, and lemmas in each of the CYP-LEX age bands, as well as the average number of tokens, types, and lemmas per book in each age band.^4^ The distribution of tokens and types across the books and age bands is depicted in Figure 1. These data show that an average book in the 10–12 age band is almost twice as long as an average book in the 7–9 age band and that the 13+ age band is the largest of the three subcorpora in terms of both the number of tokens and the number of types. Interestingly though, Table 1 also shows that, while an average book in the 13+ age band contains twice as many unique words as an average book in the 7–9 age band, the books in the 7–9 age band contain half of all the types in CYP-LEX, underscoring the richness of vocabulary in books for children in the first half of primary school.

**Table 1.** Size of the CYP-LEX age bands.

Statistic	CYP-LEX age band		
	7–9	10–12	13+
*N* tokens	11,162,653	21,837,794	37,286,770
*Mean N* (*SD*) tokens per book	27,907 (19,212)	54,694 (24,012)	93,217 (57,718)
*N* types	52,851	70,945	90,980
*Mean N* (*SD*) types per book	3,028 (1,452)	4,713 (1,550)	6,447 (2,368)
*N* lemmas	38,046	51,818	67,444
*Mean N* (*SD*) lemmas per book	2,319 (1,067)	3,520 (1,134)	4,866 (1,695)

CYP-LEX: Children and Young People’s Books Lexicon.

Each age band includes data from 400 books.

CYP-LEX is based on a corpus that is substantially larger than that used for any existing lexical database of children’s books (Masterson et al., 2010; Thompson & Sealey, 2007; Wild et al., 2013) and can thus provide more precise lexical statistics than those that are currently available. In the remainder of this section, we describe the statistics made available in CYP-LEX before turning to a discussion of the words that CYP-LEX contains. We start by discussing in what ways these words are different from those that children and young people encounter in the tele-vision programmes they watch and what these differences reveal about book vocabulary. We follow this discussion with a brief overview of the differences and similarities in book vocabulary across the age bands. Finally, in the Discussion section, we provide a more in-depth review of our findings and their potential implications for the develop-ment of reading expertise.

### Information available in the CYP-LEX database

#### Word frequencies

For each age band, each word’s most likely lemma, raw and Zipf frequency can be found in files “main_cyplex79.csv” (7–9 age band), “main_cyplex1012.csv” (10–12 age band), and “main_cyplex13.csv” (13+ age band) on the OSF site of the project. In addition, for each word in each age band, we also report its raw and Zipf frequency in the other age bands, CPWD, and SUBTLEX-UK subcorpora (van Heuven et al., 2014).

SUBTLEX-UK is arguably the best available lexical database for British English, with lexical statistics derived from subtitles on nine free-to-air television channels owned by the British Broadcasting Corporation (BBC), broadcast over 3 years (2010–2012). Seven of these channels are channels targeting adult viewers (BBC1–BBC4, BBC News, BBC Parliament, BBC HD) and two channels (Cbeebies, CBBC) are aimed at children aged 6 and under (Cbeebies) and children aged 6–12 (CBBC). Upon releasing their database, van Heuven et al. (2014) introduced a new standardised word frequency measure, the Zipf scale, a logarithmic scale going from about 1 (very-low-frequency words) to 7 (function words such as “the” or verb forms like “have”). The Zipf frequency supersedes frequency measures such as the log-transformed frequency per million (*fpmw*) or the raw frequency count because it does not take on negative values, has a straightforward unit ( $\log_{10}\;$ of the frequency per billion words), and its interpretation does not depend on the size of the corpus (e.g., in adult corpora, low-frequency words tend to have Zipf values of or below 2.5 and high-frequency words tend to have Zipf values above 4.5, see van Heuven et al., 2014). Another advantage of the Zipf scale is that it permits computation of frequency for words which are not observed in a corpus. To avoid taking the logarithm of 0, the Laplace transformation is applied (by adding 1 to the frequency count; Brysbaert & Diependaele, 2013). The Zipf scale also corrects for the number of types in the corpus such that the final formula is as follows:

(1) $$Zipf=\left(\frac{raw\;frequency\;count+1}{N\;tokens\;in\;millions\;\;+\;\;N\;types\;in\;millions}\right)+3.0\;$$

**Figure 2.**
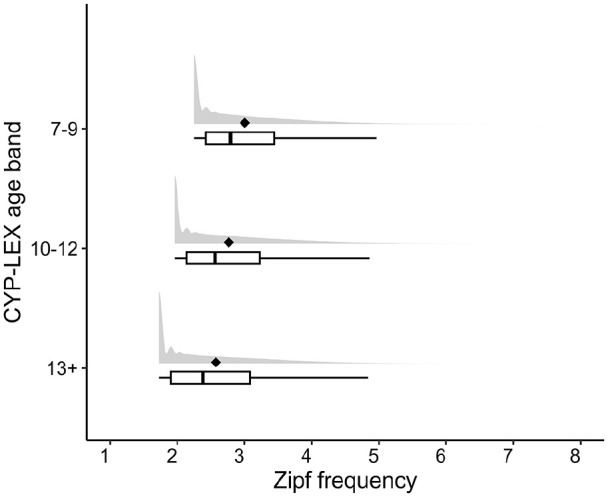
Distribution of Zipf scores across the age bands. For each age band, the black diamonds represent the means, the boxes show the lower and the upper quartiles, the black vertical lines inside the boxes represent the medians, and the horizontal lines extending from the boxes (whiskers) indicate variability outside the upper and lower quartiles.

Approximately 80% of words in the SUBTLEX-UK database have Zipf values below 3 and thus appear less than once per million words. The CYP-LEX data show a very similar pattern: 60% (7–9 age band), 67% (10–12 age band), and 73% (13+ age band) of words have a Zipf frequency of less than 3 (see Figure 2). Only about 2% (7–9 age band), 1% (10–12 age band), and 1% (13+ age band) of words have a Zipf frequency greater than 5 (more than 100 per million) and thus appear frequently. In fact, in each age band, a substantial number of words only occur once: 23% (Zipf = 2.25), 22% (Zipf = 1.96), and 31% (Zipf = 1.73) in the 7–9, 10–12, and 13+ age bands, respectively (note that the slight differences in Zipf values are due to the subcorpora having different sizes^5^). The CYP-LEX database thus shows a pattern very similar to that observed in the CPWD and SUBTLEX-UK databases, with frequency distributions skewed towards low frequencies. However, the differences across the CYP-LEX age bands also suggest that, as children grow older, the number of high-frequency words in books that they read tends to decrease, while the number of low-frequency words increases (Figure 2).

#### Part-of-speech dependent frequencies

For each word in each age band, we also report all parts of speech that this word occurs in, along with its part-of-speech-dependent frequencies (raw counts) and part-of-speech-dependent lemmas (see files “main_cyplex79.csv”, “main_cyplex1012.csv”, “main_cyplex13.csv”). For instance, in the 7–9 age band, the word “rear” occurs a total of 276 times, out of which 159 times it was classified as an adjective (JJ), 102 times as a single noun (NN), 12 times as an infinitive (VB), and 3 times as an inflected form of a verb (non-3rd ps. sg., or VBP). In all cases, the associated lemma is “rear”; however, knowing the part of speech associated with each occurrence of this word allows the researcher to distinguish between its different meanings and senses (adjective “rear”: something located at the back as in “rear window”; noun “rear”: the back part of something as in “the rear of the house”; verb “to rear” meaning “to bring up and care for a child or an animal” as in “rearing a calf”). By contrast, different lemmas are associated with the word “puzzled”: where it is used as an adjective (216 times out of 394), the associated lemma is “puzzled”, but where it is used as a verb (either in past tense or as past participle; 178 times out of 394), the lemma is “puzzle”. Thus, the part-of-speech dependent frequencies provide highly relevant information on the role in which a given word form is used in a sentence and on how often it is used in that role (see Brysbaert et al., 2012; Kuperman et al., 2012, for examples of how this information can be used in psycholinguistic research).

#### Contextual diversity

It has been argued that the number of contexts in which a word occurs (count-based contextual diversity) is a superior measure of lexical experience than the number of times it is repeated (frequency) (Adelman et al., 2006; McDonald & Shillcock, 2001). Indeed, contextual diversity has been shown to outperform word frequency in predicting lexical decision and word-naming latencies in adults (e.g., Adelman & Brown, 2008; Brysbaert & New, 2009; Yap et al., 2011) and in children (e.g., Perea et al., 2013). One reason for this finding is that high-frequency words may be very common in a subset of texts (e.g., names of characters or specific objects relevant in a specific narrative) but quite rare in other texts, meaning that readers may not encounter them at all (e.g., Cevoli et al., 2021). In CYP-LEX, we measured contextual diversity across both sentences and books such that, for each word in each age band, we report the number (or percentage) of sentences and the number (or percentage) of books in which this word appears (see files “main_cyplex79.csv”, “main_cyplex1012.csv”, “main_cyplex13.csv”). Following SUBTLEX-UK, both numbers and percentages are reported on raw (counts) and log-transformed scale. We note that there are other ways of assessing contextual diversity: for example, it has been proposed that contextual diversity measures should also consider the semantic diversity of contexts. However, as there is currently no consensus in the literature as to how semantic diversity should be calculated, with different research groups proposing different definitions of this concept (e.g., Cevoli et al., 2021; Hoffman et al., 2013; Hsiao & Nation, 2018; Johns, 2021; Johns et al., 2020; Jones et al., 2017), we refrain from prioritising either of these methods over the others.

An examination of contextual diversity across the CYP-LEX age bands revealed that, on the sentence level, the age bands appear similar, with less than 1% of all words appearing in more than 1% of sentences. On the book-level, contextual diversity is also very low: on average, each word in each age band is encountered in 7% ( σ=14% ; 7–9 age band), 7% ( σ=15% ; 10–12 age band), and 6% ( σ=16% ; 13+ age band) of books within that age band. Overall, only 13% (7–9), 15% (10–12), and 17% (13+) of types in each age band are encountered in more than 10% (*N* = 40) of books. These results are in line with our finding that word frequency distributions are skewed towards low frequencies, and indicate that most words in our corpus are encountered only in a very small subset of books. These results also suggest that books that children and young people read vary substantially in terms of which words they use, but that this variability tends to decrease with book target age, so that, in books for older children, low-frequency words are present in a higher proportion of books than they are in books for younger children.

#### Term-document matrix

In addition to contextual diversity measures described above, for each age band, we also provide a term-document matrix (in three separate files, “tdm_cyplex79.csv”, “tdm_cyplex1012.csv”, and “tdm_cyplex13.csv”). A term-document matrix represents the relationship between terms and documents in a corpus, and this relationship can be expressed in different ways. In the case of CYP-LEX, terms are defined as lemmas that appear in a particular age band (in rows) and documents are defined as individual books in that age band (in columns). We chose to express the relationship between the lemmas and the books by means of a term frequency-inverse document frequency (tf-idf) statistic (e.g., Manning et al., 2008; Rajaraman & Ullman, 2011; Robertson, 2004). The main advantage of the tf-idf statistic is that it weights the words according to how unique they are to an individual document as compared with the corpus as a whole. Crucially, the inverse frequency is used to ensure that words frequent in all documents (e.g., function words such as “of”, “as”, and “the”) are not entirely ignored, but that their impact is minimised, while infrequent terms are upweighted. Mathe-matically, for each term t , this is achieved by taking a logarithm of the ratio of the total number of documents in the corpus n to the document frequency df(t) of this term (i.e., the number of documents that contain t ), as shown below in equation (2):

(2) $$idf(t)=\log\left(\frac{\left(n+1\right)}{\left(df(t)+1\right)}\right)+1\;$$

In the formula above, 1 is added to both the numerator and the denominator to prevent zero divisions (as not every term is present in every document). The tf-idf statistic is then computed by multiplying the term’s raw frequency in a given document tf(t,d) by this term’s inverse frequency, as shown below in equation (3):

(3) tf−idf(t,d)=tf(t,d)⋅idf(t)

**Figure 3.**
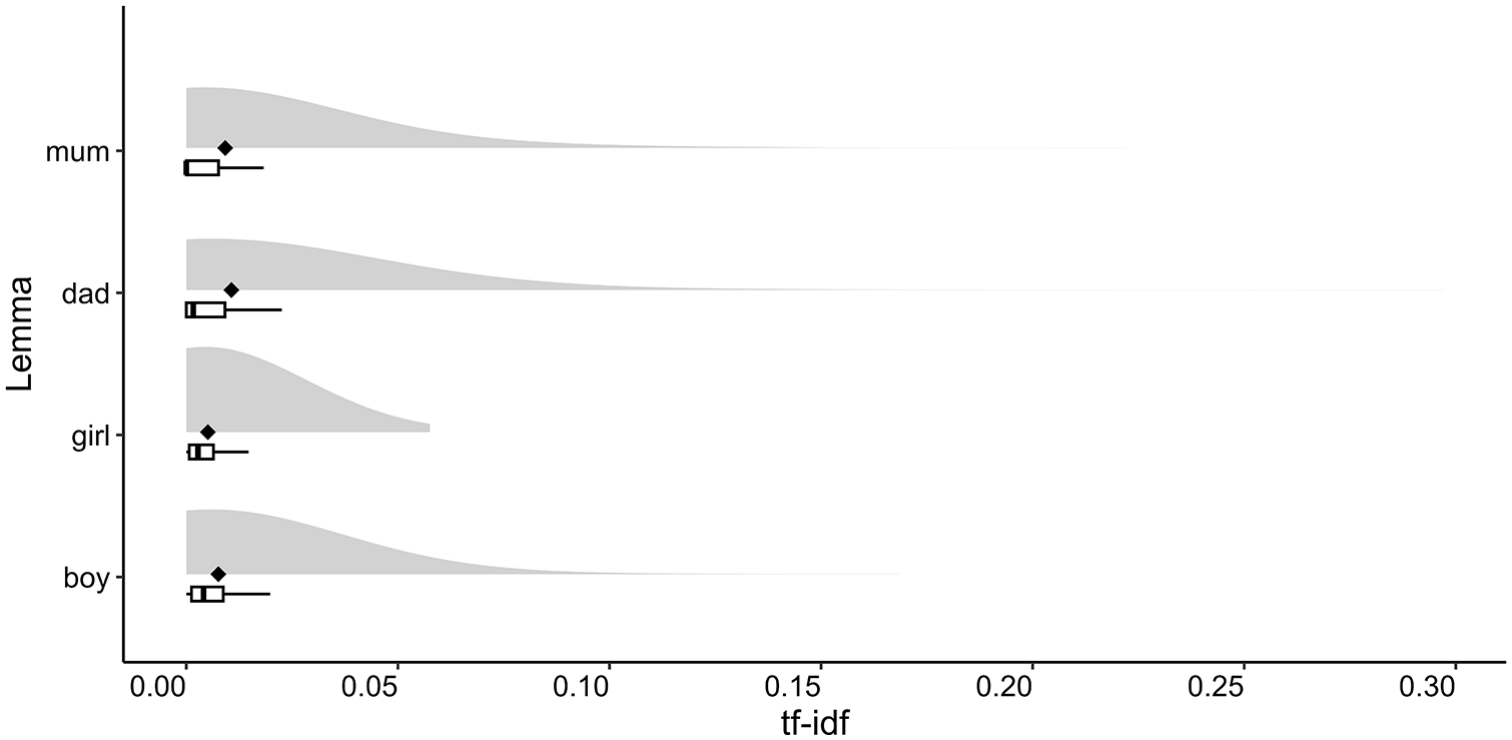
Term frequency-inverse document frequency (tf-idf) for lemmas “mum”, “dad”, “boy”, and “girl” across the books in the 7–9 age band. For each lemma, the graph shows the density plot (the “clouds”), the mean tf-idf values (the black diamonds), and the interquartile range (the boxes signify the upper and the lower quartiles, and the horizontal lines represent the data outside the upper and lower quartiles), with vertical black lines representing the median.

Because the tf-idf statistic factors in the overall frequency of a word, it provides a good measure of that word’s relevance in each individual document. For instance, in a given book, functors such as articles and prepositions may be the most common words; however, it would be incorrect to assume that these words are also the most relevant words in that book. This is because functors are typically the most common words in many, if not all, other books in a corpus, and this will be reflected in their tf-idf values, but not in their overall frequency or frequency per book values. By contrast, if a subset of books has particularly high tf-idf values for a particular word, we can infer that this word is more prevalent in these books than in any other book in the corpus. By computing the distributions of tf-idf values across the books, we can thus gauge how important, on average, a given lemma is in books targeting a specific age band, providing unique insight into the vocabulary used in children’s books. For example, if we compare how lemmas “mum”, “dad”, “boy”, and “girl” are used in books included in the 7–9 age band, we can conclude that, on average, “dad” is weighted as more important than “mum”, and “boy” as more important than “girl” (Figure 3). However, this figure also shows that these differences in means are most likely driven by a small number of books prioritising one lemma over the other, and may not reflect how these lemmas are used in most books in this age band.

### Features of book vocabulary in the CYP-LEX database

#### Words in books versus on television

To examine how child-ren’s book vocabulary differs from that on television, we inspected (1) how many words from the CYP-LEX database are also present in the SUBTLEX-UK database, and (2) for words that are encountered both in CYP-LEX and SUBTLEX-UK, how CYP-LEX word frequencies compare to those in SUBTLEX-UK.

Although SUBTLEX-UK is largely a database of adult word frequencies, it also includes words derived from subtitles on two children’s channels, Cbeebies, targeting pre-school children, and CBBC, designed for children between 6 and 12 years. Overall, the SUBTLEX-UK database contains 159,235 types (derived from 201,335,638 tokens) and is thus substantially larger than CYP-LEX (105,694 types, derived from 70,287,217 tokens). However, its Cbeebies (27,236 types, derived from 5,848,083 tokens) subcorpus is significantly smaller than either of the CYP-LEX age bands, while the CBBC (58,691 types, derived from 13,612,278 tokens) subcorpus is comparable in size only to the 7–9 age band (52,851 types, derived from 11,162,653 tokens). The full textual content of the SUBTLEX-UK corpus (i.e., tokens) is not publicly available due to copyright issues, and, consequently, the differences in the size of the CYP-LEX and SUBTLEX-UK corpora pose challenges for direct comparisons between the resulting databases. To minimise the potential impact of these size differences, we combined the Cbeebies and the CBBC subcorpora, which resulted in a list of 63,081 types (derived from 19,460,361 tokens). We then examined how many words in the 7–9 and 10–12 age bands are not included in this combined word list. Regarding the first comparison, because the combined subtitle list is larger than CYP-LEX 7–9 (in terms of the number of both types and tokens) and includes words encountered in television programmes for older children (up to the age of 12), we reasoned that the presence or absence of CYP-LEX 7–9 words in this combined list would be informative regarding the differences in vocabulary in books versus on television. Following this logic, we then also examined how many words in each of the CYP-LEX age bands are not present in the entire SUBTLEX-UK database. Regarding the comparison between the 10–12 age band and the combined Cbeebies and CBBC list, we report this analysis for completeness but we acknowledge that these figures should be treated with caution given that the 10–12 age band was derived from a slightly larger corpus (a difference of 2.4 million tokens) than that from which the Cbeebies and the CBBC lists were derived.

This analysis revealed that children’s books contain many words that are never encountered on television. For instance, 28% (*N* = 14,873) of words in books for 7- to 9-year-olds never appear in age-appropriate BBC television programmes and programmes for older children. Most of these words are nouns (57%), followed by verbs (18%) and adjectives (12%). While the majority of these words (90%) are encountered less than 10 times (Zipf frequencies below 3), 2% occur 50 times or more (Zipf frequencies between 3.5 and 5.5). Likewise, 40% (*N* = 28,533) of words in the 10–12 age band are not encountered in the combined Cbeebies and CBBC list. Finally, while the SUBTLEX-UK database as a whole contains most of the words (91%) in the youngest age band, 14% (*N* = 10,231) and 21% (*N* = 19,472) of words in the two older age bands are missing from SUBTLEX-UK. SUBTLEX-UK includes content from all television programmes broadcast on the BBC over 3 years (2010–2012) and thus represents a comprehensive record of British television language; therefore, the fact that children’s books include so many words that are missing from SUBTLEX-UK is remarkable. About 90% of book words not included in SUBTLEX-UK are encountered less than 10 times (Zipf frequencies below 2.5); however, 2% occur 50 times or more (Zipf frequencies between 3 and 5). Inspection of these unshared words suggests that many are morphologically complex (e.g., “conquerable”, “unprocurable”, “sorrowfully”). This impression was further supported by an examination of the morphological structure of words missing from SUBTLEX-UK as documented in the MorphoLex database (Sánchez-Gutiérrez et al., 2018). MorphoLex comprises morphological information for 68,624 words from the English Lexicon Project (Balota et al., 2007); 19% (*N* = 5,257) of CYP-LEX words missing from the SUBTLEX-UK database have entries in the MorphoLex database, and 77% (*N* = 4,052) of these words are morphologically complex.

For those words that are present both in CYP-LEX and SUBTLEX-UK, Table 2 reports the correlations between these words’ frequencies in each of the CYP-LEX age bands and their frequencies in each of the SUBTLEX-UK subcorpora. The Hotelling–Williams test for differences in correlations that are themselves intercorrelated (Steiger, 1980) showed that frequencies from the 7–9 age band correlated more strongly with those from CBBC than with those from the other two SUBTLEX-UK subcorpora (CBBC vs. Cbeebies: t=47.85 , p<.001 ; CBBC vs. adult: t=38.25 , p<.001 ). For the other two CYP-LEX age bands, we observed higher correlations with the adult subcorpus of SUBTLEX-UK than with either Cbeebies (10–12: t=67.80 , p<.001 ; 13+: t=105.36 , p<.001 ) or CBBC (10–12: t=7.94 , p<.001 ; 13+: t=52.51 , p<.001 ). The fact that word frequencies in the 10–12 age band correlate more strongly with those in the adult SUBTLEX-UK subcorpus than with those in the CBBC subcorpus is particularly striking, and suggests that the way that words are used in children’s books may be more sophisticated than the way they are used on children’s television.

**Table 2.** Correlations between CYP-LEX frequencies and those from SUBTLEX-UK.

CYP-LEX age band	SUBTLEX-UK subcorpus		
	Cbeebies	CBBC	Adult
7–9	*r* = .674	*r* = .773	*r* = .724
	*N* = 20,485	*N* = 36,823	*N* = 48,077
10–12	*r* = .626	*r* = .749	*r* = .757
	*N* = 21,236	*N* = 40,972	*N* = 60,714
13 +	*r* = .578	*r* = .716	*r* = .765
	*N* = 21,649	*N* = 43,455	*N* = 71,508

CYP-LEX: Children and Young People’s Books Lexicon; SUBTLEX-UK: Word frequency database for British English derived from subtitles used on British television; CBBC: Children’s BBC television channel.

In each cell, the top row shows the Pearson’s correlation coefficient and the bottom row shows the number of words shared between the SUBTLEX-UK subcorpora and the CYP-LEX age bands. For all correlations, p<.001 .

#### Words across the age bands

The aim of the analysis reported in this section was to understand the similarities and differences in how words are used across the age bands. To this end, we first examined whether the frequencies of the words that were encountered in more than one age band were alike across the age bands. 45,318 words (86%) and 47,426 (90%) words from the 7–9 age band were also present in the 10–12 and 13 + age bands, respectively, whereas 59,942 words (84%) from the 10–12 age band were also present in the 13+ age band. The frequencies of the shared words were highly correlated (all p<.001 ): r=.877 for the frequencies in the 7–9 and 10–12 bands, r=.823 for the frequencies in the 7–9 and 13+ bands, and r=.871 for those in the 10–12 and 13 + bands. The Hotelling–Williams test showed that the frequencies in the 7–9 age band were more strongly correlated with those in the 10–12 age band than with those in the 13+ age band ( t=48.29 , p<.001 ), and that the frequencies in the 10–12 age band were more strongly correlated with those in the 7–9 age band than with those in the 13+ age band ( t=5.23 , p<.001 ). This result suggests that, for words that are shared across the age bands, their frequency of use in books for 10- to 12-year-olds is more similar to that in books for younger children than it is to that in books for older children.

**Figure 4.**
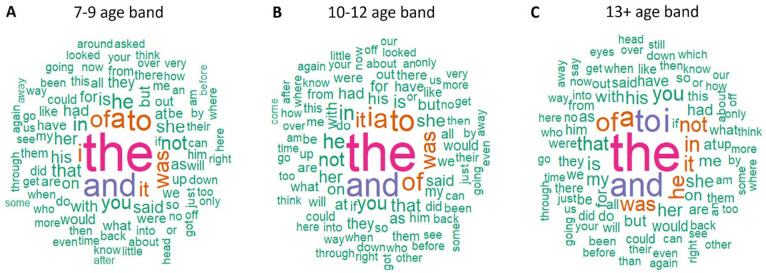
100 most common words in each age band. Words are colour- and size-coded with respect to their Zipf frequency: green for Zipf < 7.13, orange for 7.13 < Zipf < 7.42, purple for 7.42 < Zipf < 7.50, and pink for Zipf > 7.50, with greater font size representing higher frequency within each of these ranges.

Next, we examined which words in the corpus were used most frequently and whether the age bands differed in terms of their most common words. The age bands appear very similar in terms of their 100 most frequent words (Zipf frequencies between 6 and 7.75; see Figure 4). Similar to lexical databases derived from other corpora (e.g., CPWD, SUBTLEX-UK), these words amount to about half of all tokens (54%) in each age band. Most of these are function words (approx. 70%) such as prepositions (e.g., “in”, “with”; 18%), personal pronouns (e.g., “I”, “he”; 17%), auxiliary verbs (e.g., “be”, “are”; 14%), determiners (e.g., “a”, “the”; 7%), and coordinating conjunctions (e.g., “and”, “but”; 3%), but the list also includes a small proportion of adverbs (e.g., “again”, “away”, “back”; 14%), adjectives (e.g., “little”, “right”; 4%), nouns (e.g., “time”, “way”; 3%), and lexical verbs (e.g., “go”, “know”, “think”, “like”, “see”; 6%). Thus, a breakdown of word class in the 100 most common words in the CYP-LEX corpus very closely resembles that in the CPWD, in which 89% were classified as function words (note that, in Masterson et al., 2010, adverbs and verbs such as “say”, “ask”, “look”, and “like”—termed “verbs with general meaning”—were treated as function words).

**Figure 5.**
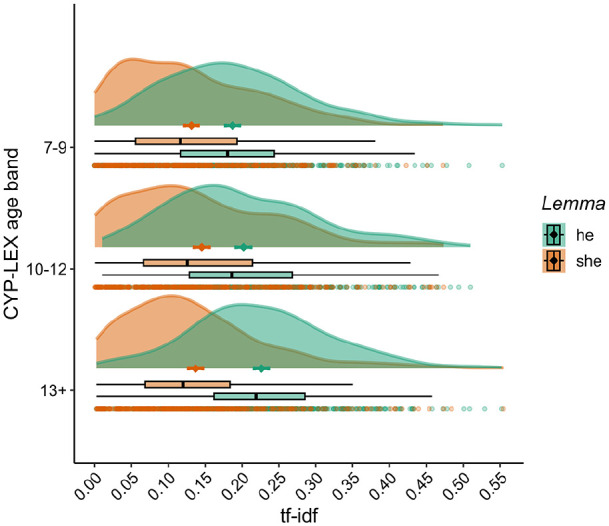
Term frequency-inverse document frequency (tf-idf) for lemmas “he” and “she” across books and age bands. For each lemma and age band, the graph shows the density plot (the “cloud”) with the mean (the diamond) and two standard errors of the mean (error bars), the individual tf-idf scores within that age band (the dots), and the interquartile range, with vertical black lines representing the median.

Among the top 100 words, the most pronounced differences across the age bands pertain to the use of personal pronouns, which, in each age band, amount to around 17% of the 100 most common words. The pronoun “I” is present in every band and its frequency increases as a function of the books’ target age—ranked 6th (7–9 age band), 5th (10–12 age band), and 3rd (13+ age band). It is thus more common than function words such as “a” or “to”, possibly indicating a growing self-focus in books targeting teenagers as opposed to younger children. The pronoun “he” is also very common in each age band, ranked 9th, 10th, and 7th in the three age bands, respectively. Intriguingly, “she” is used much less often—14th in the 7–9 and 10–12 age bands, and 17th in the 13+ age band—with the gap between “he” and “she” widening as the books’ target age increases. This pattern also holds at the lemma level, with the lemma “he” (ranked 4th in the 7–9 and 13+ age bands, and 6th in the 10–12 age band) being used more frequently than the lemma “she” (ranked 9th in the 10–12 and 13+ age band, and 8th in the 7–9 age band). Moreover, the distribution of tf-idf scores for these two lemmas across the individual books (Figure 5) demonstrates that, on average, books in each age band tend to use the lemma “he” more than the lemma “she”, with this difference being particularly large in the 13+ age band. Taken together, these results suggest that children’s books tend to focus more on male than on female characters and that, as the books’ target age increases, this trend continues on the upward trajectory.

Regarding the plural forms of personal pronouns, the frequency of both first-person and third-person plural pronouns does not seem to vary across the age bands. However, in each age band, third-person plurals are used more frequently than first-person plurals, as indexed by higher frequency of the lemma “they” as compared with that of the lemma “we” in terms of both raw counts and tf-idf scores. Because first- and third-person plurals are often interpreted as markers of group identity (e.g., Pennebaker & Lay, 2002), this pattern could indicate that membership in social groups is an important concept in children’s literature and that, regardless of the readers’ target age, the characters are more often described as not belonging than belonging to a group. Given the high pre-valence of “I” in the books (and particularly so in books written for young people), one could further speculate that the book characters’ self-categorisation could be based on personal rather than on social identity. We note, however, that this preliminary interpretation needs further evaluation and that our data do not speak of the uniqueness of this pattern to children’s books, nor can they inform on the extent to which this pattern may apply to other language registers (e.g., spoken language and/or adult literature).

**Figure 6.**
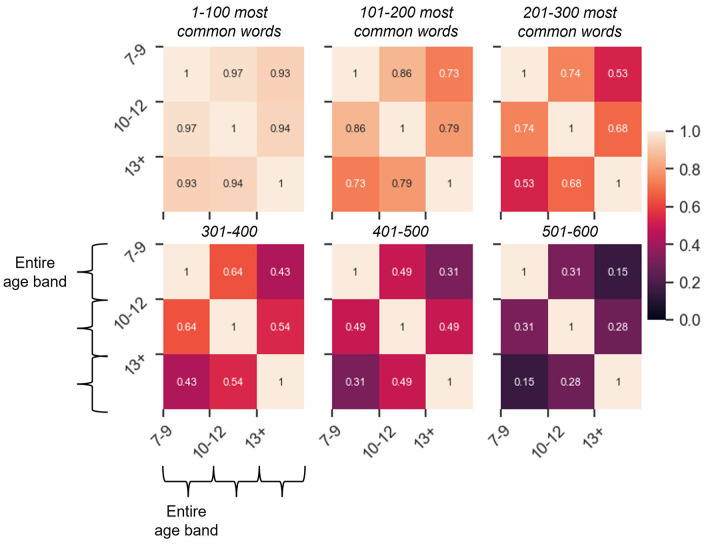
Similarity between the 600 most common words in the three age bands. The 3 × 3 matrices represent how similar the age bands are in terms of 600 most common words, with one matrix per 100 words, presented from left to right and from top to bottom in descending order (words 1–100, words 101–200, words 201–300, etc.). The amount of overlap across the sets is represented through variation in colour, and the numbers in the cells show the proportion of shared words.

Beyond the top 100 words, our analysis shows that the similarity of vocabulary across the age bands decreases as a function of word frequency (Figure 6). Indeed, while the first hundred of the top 600 words are almost identical across the age bands (93%–97% overlap), the amount of overlap is reduced to 73%–86% for the second hundred and to 53%–73% for the third hundred. By the time the sixth hundred most common words (words 501–600) are reached, the overlap between the 7–9 and the 13+ age bands is reduced to 15% and that between the 7–9 and the 10–12 age bands to 31%. Interestingly, for each set of 100 words among the top 600 words, the overlap between the 7–9 and the 10–12 age bands is greater than that between the 7–9 age band and the 13+ age band, suggesting that, with respect to the most common words, books for child-ren over 13 are less like those in the two younger age bands than books in these age bands are to each other. This pattern was also observed for frequency correlations for words shared across the age bands.

Our analysis further showed that the decrease in similarity across the top 600 words was accompanied by a decrease in the proportion of function words: for instance, among the second hundred of the top 600 words, only about 43% are functors, and, among the third hundred, no more than 38%. Notably, despite their preponderance in the corpus, function words account for only a very small percentage of types: in each age band, 57%–59% of all types are nouns, 20%–22% are verbs, followed by adjectives (14%), adverbs (4%), proper nouns (3%–4%), and foreign words (1%), with all other parts of speech accounting for less than 0.5% of all types. These findings indicate that the most significant vocabulary differences across the age bands should be attributable to the use of words with lower frequencies and that most of these words are nouns, followed by verbs and adjectives.

Indeed, while a high percentage of words are shared across the age bands, each age band contains vast numbers of words that do not appear in the younger age band, and the vast majority of these are very low in frequency. Thus, 36% (*N* = 25,627) of words in the 10–12 age band and 48% (*N* = 43,549) of words in the 13+ age band are not present in books for children aged between 7 and 9 years, while 34% (*N* = 31,025) of words in the 13+ age band are not encountered in books for children aged 10–12 years. Strikingly, within the age bands, the vast majority of these “new” words (73%–74%) only appear 3 times or less, and only about 1% of these words are encountered more than 100 times (most of these words are names and, for the 13+ age band, swear words). It follows that about a third of these “new” words in the 10–12 and 13+ age bands (i.e., words that are never encountered in the 7–9 and 10–12 age bands, respectively) appear in a maximum of 3 books such that the readers are extremely unlikely to ever encounter them (see Supplementary material B for a list of words from bands 10–12 and 13+ that are missing in bands 7–9 and 10–12, respectively; https://doi.org/10.17605/OSF.IO/SQU49).

It is important to recognise that, compared with books for adolescents, books for children who have only recently begun to read independently typically contain illustrations and have shorter sentences, fewer words per page, and fewer and shorter chapters. Indeed, in CYP-LEX, the mean book length (number of tokens) in the 10–12 age band is twice that in the 7–9 age band, while an average book in the 13+ age band is 1.7 times longer than that in the 10–12 age band (see Table 1). In corpus linguistics, it is customary to control for differences in corpus size by equating the corpora in terms of the number of tokens (e.g., by taking random samples of the size of the smaller corpus). Within the context of our work, this approach would result in si-mulating an unrealistic scenario, whereas equating the number of books included in each age band is a more ecologically valid approach to quantifying children’s reading experience. We acknowledge that, under this approach, it is not possible to distinguish whether the differences in book vocabulary across the age bands are driven solely by differences in their lexical content or are at least to some extent a result of differences in book length, and, therefore, we do not make any inferences regarding this matter. Rather, we take our findings to indicate that, with about 40% of words in each age band having a raw frequency of 3 or less and about half of the words a raw frequency of 6 or less, a child wishing to enhance their vocabulary and move beyond the most common words would need to read widely. Furthermore, the results reported above also suggest that even for those who do read widely, understanding the vocabulary used in books still poses a challenge, with many new words to work through in age-appropriate books, and even more so as readers transition to books aimed at older children.

**Figure 7.**
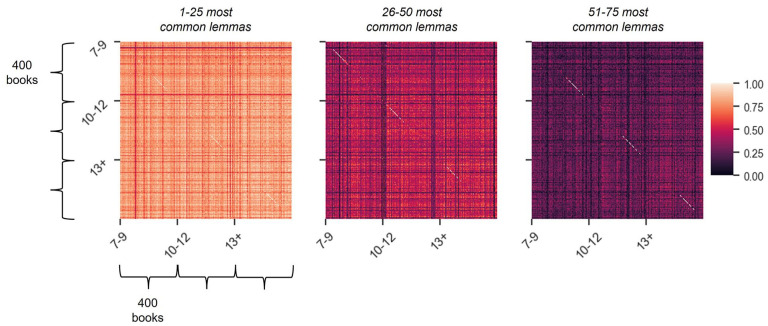
Similarity between the 75 most common lemmas in the three age bands. The three 1,200 × 1,200 matrices show how similar the books within the age bands (400 books per age band) are to one another in terms of their 75 most common lemmas, with one matrix per 25 lemmas, presented from left to right in descending order in terms of frequency (lemmas 1–25, 26–50, and 51–75). Each cell represents the proportion of lemmas in one book that is shared with every other book in the corpus, with less overlap reflected in darker colours.

Thus far, we have shown that book vocabulary for younger primary school children is comparable to that for older children and young people regarding the first few hundred most common words, but that the age bands differ substantially in terms of words with lower frequencies. Our next analysis showed that this pattern of results holds also at the level of individual books. For each book in the corpus (*N* = 1,200, with 400 books per age band), Figure 7 shows the proportion of lemmas, out of the 75 most common lemmas (i.e., including function words), that each book shares with every other book in the corpus. It is immediately apparent that, within each age band, most of the books share the first 25 of the 75 most frequent lemmas. Yet, the amount of overlap reduces drastically for lemmas 26–50, and even more so for lemmas 51–75. This interpretation was confirmed through a statistical test which was conducted by first computing a mean vector for each age band and set of lemmas and then comparing these mean vectors by means of a *t*-test (Table 3). It is of note that, for a book of an average size, 25 lemmas amount to about 1% (7–9 age band), 0.7% (10–12 age band), and 0.5% (13+ age band) of all the lemmas it contains. Consequently, these results suggest that the number of lemmas that can be expected to occur consistently in books written for children of the same age is extremely low (no more than 1%), with the vast majority of these lemmas being function words.

**Table 3.** Overlap across the 75 most common lemmas per book across the age bands.

CYP-LEX age band		Lemmas 1–25	Lemmas 26–50	Lemmas 51–75
7–9		*µ* = .747, σ=.091	*µ* = .387, σ=.124	*µ* = .215, σ=.095
	Lemmas 26–50	t=935.720 , p<.001		
	Lemmas 51–75	t=1,617.002 , p<.001	t=438.703 , p<.001	
10–12		*µ* = .791, σ=.077	*µ* = .434, σ=.140	*µ* = .242, σ=.100
	Lemmas 26–50	t=892.000 , p<.001		
	Lemmas 51–75	t=1,743.001 , p<.001	t=444.621 , p<.001	
13+		*µ* = .813, σ=.074	*µ* = .462, σ=.132	*µ* = .265, σ=.104
	Lemmas 26–50	t=926.472 , p<.001		
	Lemmas 51–75	t=1,707.723 , p<.001	t=467.578 , p<.001	

CYP-LEX: Children and Young People’s Books Lexicon.

For each age band, the top row shows the mean (μ) and the standard deviation (σ) for proportion of overlap in 1–25, 26–50, and 51–75 most common lemmas across the books. The bottom two rows show the results of a *t*-test comparing the average amount of overlap across the sets of lemmas (1–25, 26–50, 51–75), separately for each age band.

**Figure 8.**
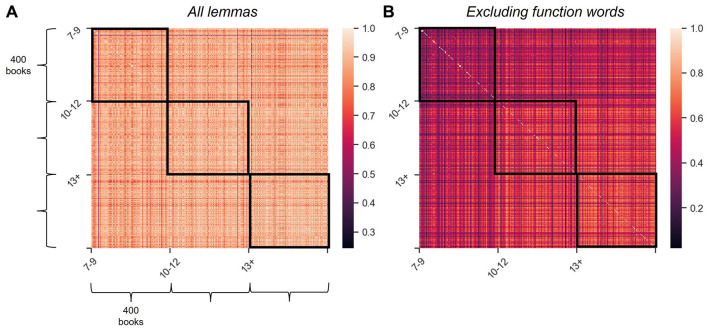
Cosine similarity across the books using all lemmas in the corpus (panel A) and all lemmas excluding 185 function words as defined in the NLTK Python library (panel B). The cells record cosine similarity of each of the 1,200 books in the corpus with every other book, with darker colours representing lower similarity. Black squares highlight the cosine similarity scores within the age bands.

To examine the similarity of vocabulary within the age bands beyond the most common lemmas, we built a document-term matrix, where each row corresponded to a book (*N* = 1,200) and each column corresponded to each unique lemma observed in the entire CYP-LEX database (*N* = 75,386). Each cell of the matrix recorded the raw frequency of each unique lemma in each book (a value of zero was recorded if a lemma did not occur in a book). This approach resulted in each book being represented in the form of a vector of values representing the frequency of each lemma in the database in this particular book. We then measured the similarity between all individual vectors (books) by computing their cosine similarity (i.e., their inner product space) and then repeated this process while excluding those lemmas that corresponded to function words.^6^ An important advantage of this analysis is that it is not influenced by differences in book length. Figures 8A and 8B visualise the resulting similarity matrices in the form of two heatmaps, and the results of a statistical analysis comparing the similarity scores across the age bands are reported in Table 4. Both of these analyses revealed that the similarity between books in the 7–9 age band is much lower than the similarity between books in the 10–12 and 13+ age bands. Figure 8B clearly shows that, in contrast to the 7–9 age band, where most books appear to have low similarity to one another, only a small subset of books that children aged 13 + read differ from other books regarding their vocabulary. The majority of these “less similar” books are books listed in the GCSE syllabus for English. It is therefore likely that these books’ low similarity to other books in the 13+ age band is due to the fact that they were written long ago when language was used quite differently. Summing up, our findings indicate that, apart from some very frequent (function) words, books for younger primary school children differ more substantially from each other with respect to both which words they use and how often they use them. These differences are attenuated in the literature targeting older readers, suggesting a substantially higher degree of lexical homogeneity in books read by children in the final years of primary school and in secondary school.

**Table 4.** Similarity of books within the age bands for all lemmas (top panel) and excluding function words (bottom panel).

		CYP-LEX 7–9	CYP-LEX 10–12	CYP-LEX 13 +
All lemmas		*µ* = .859, σ=.071	*µ* = .882, σ=.059	*µ* = .885, σ=.066
	10–12	t=−100.334 , p<.001		
	13 +	t=−109.085 , p<.001	t=−14.587 , p<.001	
Excluding function words		*µ* = .429, σ=.124	*µ* = .524, σ=.132	*µ* = .603, σ=.135
	10–12	t=−209.383 , p<.001		
	13 +	t=−378.028 , p<.001	t=−167.095 , p<.001	

CYP-LEX: Children and Young People’s Books Lexicon.

For each comparison, the top row shows the mean (μ) and the standard deviation (σ) for cosine similarity across the books, separately for each age band. The two bottom rows report the results of a *t*-test comparing the mean cosine similarity vectors across the age bands. For each age band, a mean vector was derived by averaging across all 400 vectors (one for each book) within that age band.

## Discussion

This article presents a new database of lexical statistics derived from 1,200 books popular with British children aged between 7 and 16 years, equally distributed across three age bands (7–9, 10–12, and 13+). CYP-LEX surpasses its predecessors (e.g., CLLIP, CPWD, OCC) in terms of its size, the breadth of the age range that it covers, and in the fact that it is publicly available. CYP-LEX provides a wealth of information about the vocabulary that children and young people are exposed to when they read independently: for each word in each age band, the main files (“main_cyplex79.csv”, “main_cyplex1012.csv”, “main_cyplex13.csv”) contain frequency statistics (raw count, Zipf score, PoS-dependent frequencies), associated lemmas (most likely lemma and PoS-dependent lemmas), and count-based contextual diversity information (number and percentage of sentences and books in which a word was encountered), and the additional files report term-do-cument matrices with tf-idf values (“tdm_cyplex79.csv”, “tdm_cyplex1012.csv”, “tdm_cyplex13.csv”). Thus, CYP-LEX offers unprecedented insight into the nature of children’s book language, while opening countless new research possibilities in the study of reading acquisition and development. In the following, we discuss initial findings derived from CYP-LEX regarding child-ren’s book vocabulary and their potential implications for understanding children’s reading.

### Children may encounter many unfamiliar words in books

One of the most striking findings from our initial analyses of CYP-LEX is that a large percentage of words in the books read by British children may not occur on British television. Our analyses show that 28% of words in the 7–9 age band and 40% of words in the 10–12 age band are not present in the SUBTLEX-UK sample of Cbeebies and CBBC programming, which are aimed at children up to the age of 12. Most of these words are encountered rarely; however, 2%–3% of these words occur at least 50 times in the relevant CYP-LEX age bands. Examples of these more common words from the 7–9 age band include names (e.g., Eugenia, Mirella), scientific and art terms (e.g., “meridian”, “homunculus”, “aria”), and morphological constructions (e.g., “guiltily”, “indignantly”, “apologetically”, “incredulously”, “bombinating”). Examples from the 10–12 age band comprise historical and political vocabulary, often of Latinate origin (e.g., “communists”, “suffragists”, “abolitionist”, “legislature”, “marquis”, “inquisitor”, “missionary”), theological concepts (e.g., “quaker”), names of famous personalities (e.g., Seneca, Hitler), and, like in the 7–9 age band, morphologically complex words (e.g., “purposefully”, “mournfully”, “bewilderment”, “disagreeable”, “deathless”).

Similarly, one-fifth of words in the 13+ age band are missing from television programmes developed for adult audiences. While 92% of these words are encountered less than 10 times in the 13+ age band, 2% occur 50 times or more. Like in the other age bands, morphologically complex words dominate. Many of these words are rarely used in spoken language (e.g., “mordicant”, “amaurotic”, “conspiratorially”, “returnees”, “demurral”, “revenant”, “licentiate”, “groggily”), while other examples include jargon (e.g., “commandant”, “proctor”, “manoeuvred”, “procurator”), and historic or political terms (e.g., “antisemitism”). It is important to recognise that television programmes also contain words that do not appear in children’s books, including rare and complex vocabulary (e.g., “abseiling”, “hooliganism”, “coalition”). Interestingly, however, visual inspection suggests that most of words encountered on te-levision but not in children’s literature are monomorphemic and colloquial (e.g., expressions typical for spoken but not written language such as “lol”, “innit”, “gimme”).

It is of note that, although television language is often used as a proxy for spoken language, important differences exist between these two language registers (e.g., television language is likely to be richer than everyday spoken language, and the majority of television language is scripted). Moreover, children may be familiar with some of the words missing from the television corpus (in particular, the swear words that feature prominently in the 13+ corpus). It is also important to acknowledge that SUBTLEX-UK was assembled from subtitles from BBC television programmes that were broadcast in 2010–2012, and it is possible that the vocabulary used on British television has undergone various changes and now resembles book vocabulary to a greater or lesser degree than it did 10 years ago. However, because books were included in CYP-LEX based on their popularity rather than their year of publication, CYP-LEX contains many books that were published more than a decade ago (i.e., at the time when SUBTLEX-UK was built) but that are still popular with British children. In addition, most of the BBC television programmes for children represented in SUBTLEX-UK are still being aired and watched today. Consequently, while our findings could be to some extent due to changes that have occurred in the English language over the past decade, we think it unlikely that they are a primary driver of the differences between book and television vocabulary that we report.

We have argued above that it is likely that children’s first experience with most of the sophisticated words encountered in CYP-LEX books would be through reading. It is widely accepted that children’s reading comprehension in the initial years of reading instruction is a product of their decoding ability and their oral language (Gough & Tunmer, 1986; Hoover & Gough, 1990). This idea is known as the *Simple View of Reading*, and it asserts that children use their phonics knowledge to decode unfamiliar printed words to sound-based representations and then compute the meanings of these words using their spoken language knowledge (e.g., “swim” → [swim], “propel the body through water using the limbs”). However, the CYP-LEX data suggest that children may be encountering a large proportion of words in books that are not in their spoken vocabulary, and further, that this occurs from the earliest years of independent reading. The fact that child-ren will be unfamiliar with many of the words encountered in books means that they will frequently need to use contextual information rather than pre-existing spoken language knowledge to infer meaning. The CYP-LEX data thus highlight the sophistication of vocabulary used in books, and they underscore both the challenge that child-ren face when they begin to engage in independent reading and the opportunity that books present for vocabulary acquisition.

### Few words used in books are encountered frequently

Half of the tokens in each of the CYP-LEX age bands comprise 100 most frequent words, a finding consistent with previous studies of children’s (e.g., Masterson et al., 2010) and adults’ (e.g., van Heuven et al., 2014) corpora. These words occur in the corpus with a frequency of 10,000 per million (Zipf frequencies between 6 and 7) and are thus so common that a beginning reader will quickly learn to recognise them without the need for decoding. This process of learning to recognise words “by sight” is known as *orthographic learning* (e.g., Castles & Nation, 2006; Nation & Castles, 2006).

Orthographic learning is thought to arise gradually through self-teaching as children begin to read independently (Share, 1995). Under this view, the acquisition of orthographic knowledge can be seen as a by-product of alphabetic decoding because decoding requires the reader to focus on how the individual letters are combined to spell a word. With repeated exposure, knowledge of the correct spelling (i.e., the *precision* of the lexical representation) and the ability to quickly switch between print-meaning combinations depending on the context (i.e., the *flexibility* of the lexical representation, e.g., reading about working in a bank versus sitting on the river bank) improve, leading to an increase in the *lexical quality* of the words’ mental representation (e.g., Perfetti, 1992, 2007; Perfetti & Hart, 2002) and thus lessening the reliance on decoding. Orthographic learning is an item-based process, such that, at any given point in time, some words may be processed effortlessly and automatically, while other, less well-known, or novel, words may require decoding (e.g., Castles & Nation, 2006; and see, e.g., Grainger et al., 2012; Pritchard et al., 2018; Ziegler et al., 2014, for computational implementations of this item-based mechanism). Therefore, for each individual word, the speed of transition from effortful to automatic reading should depend on how often this word is encountered in print (i.e., its *cumulative frequency*, see Zevin & Seidenberg, 2002).

If repeated exposure is key to orthographic learning, then the CYP-LEX data suggest that, for the top 100 words, this will occur very quickly. Critically though, most of the 100 most common words are functors that have little lexical meaning and are used primarily to express (grammatical) relationships between other words. Consequently, being able to read these words effortlessly will not be sufficient to read for meaning. Consider the following sentence, selected from a randomly-chosen book^7^ from the 7–9 corpus: “Then a mischievous thought flashed across her eyes, and she pursed her lips together and pushed her tongue forward”. If this sentence is reduced to those words that are included in the pool of the top 100 words for this age band, a reader is left with the following statement: “Then a . . . her . . ., and she . . . her . . . and . . . her”. Most emerging readers will be able to infer that this sentence most likely describes a series of actions performed by a female character; however, the unique meaning of the sentence is lost. This simple example illustrates why being able to recognise every second word in a text effortlessly is not enough to understand what this text is about.

Moving beyond the top 100 words, the proportion of other high-frequency words (Zipf frequencies of between 4 and 6) is relatively low—11% in the 7–9 age band, 9% in the 10–12 age band, and 7% in the 13+ age band. About 700–800 of these words have frequencies between 100 and 1,000 per million, and the remaining 5,100–5,300 words are encountered between 10 and 100 times per million words. There is no consensus on the number of exposures necessary for orthographic learning; however, the small percentage of high-frequency words in children’s books suggests that only a relatively small subset of types may be learned to the degree that they are recognised automatically and effortlessly.

### Most words used in books occur infrequently and in few books

The CYP-LEX data show that the vast majority of words used in this sample of children’s books are very low in frequency and that the proportion of low-frequency words increases as a function of book target age. 60% and 67% of words in the 7–9 and 10–12 age bands, respectively, have a frequency of less than 1 per million, and, in the 13+ age band, this number surges to 73%. On average, each word in each age band is encountered in 6%–7% of books within that age band. Moreover, in each age band, about a third of all words (28%–30%) appear in only one book out of 400, and most of these words (73%–75%) are only used once in this book. These figures mean that, in each age band, around one-fifth of all words occur only once. Examples include “disreputability”, “inalterable”, “reconnoitring” (13 + age band); “unwinnable”, “bolshevist”, “inhumanely” (10–12 age band); “supernaturally”, “untrustable”, and “fascist” (7–9 age band). Taken together, these figures suggest that books within the age bands vary substantially with respect to which of the less common words they use, and our analysis of book vocabulary at the lemma level (Figures 7 and 8, Tables 3–4) indicates that this lexical variability extends beyond the level of word forms, particularly in books for 7- to 9-year-olds. Thus, apart from some highly frequent lexical items, many lexemes (i.e., words related through inflection) and lemmas encountered in one book cannot be expected to be present in a different book even within the same age band. These data further underline why reading constitutes a challenge, particularly for those who have only just begun to read independently.

Our initial analysis of vocabulary across the age bands also shows why, for many children, reading *continues* to pose a challenge as they grow older. More than a third of words in the 10–12 age band are never encountered in books for younger children, and more than a third of words in books for children over 13 do not occur in books targeting children between 10 and 12. Critically, about 73% of these “new” words are encountered only a few times and in a very small subset of books. For instance, 25,627 words from the 10–12 age band are not used in books from the 7–9 age band, and 18,646 of these words (73%) do not occur in the 10–12 books more than 3 times. Examples of such words include “mistrustfulness”, “suburbanites”, “biracially”, and “optometry”—each of these words occurs once in one book in the 10–12 age band and never in any other age band.

The preponderance of low-frequency words in child-ren’s books and the fact that books written for children of the same age tend to vary greatly in the words they use underscore the importance of alphabetic decoding skills and morphological knowledge, both for reading acquisition and for the development of reading expertise. We have reported that, based on our initial analyses, CYP-LEX appears to contain a substantial number of multimorphemic words that may not be in a child’s oral vocabulary. Without the knowledge of how morphology underpins the mapping between spelling and meaning—that specific sequences of letters are associated with specific meanings (e.g., un- meaning “not”) and can alter the meanings of other sequences of letters in highly predictable ways (e.g., “unlock”, “unzip”, “untie”)—a reader will not be able to interpret these (e.g., Rastle & Davis, 2008). Likewise, because the stems of many of these morphologically complex words will be in the child’s spoken vocabulary, a solid grounding in grapheme–phoneme relationships will be needed to decode these stems and compute their meanings. To illustrate, while a child may not have encountered the low-frequency word “mournfully” previously, it should be possible to estimate its meaning by first segmenting the word into components using morphological knowledge ([mourn] + [-ful] + [-ly]) and then decoding the stem (mourn → [mɔːn], “to feel or show sorrow for the death of someone”) using phonics knowledge. Research suggests that readers become increasingly skilled at segmenting words rapidly into their morphemes (e.g., Beyersmann et al., 2012, 2015; Dawson et al., 2017), and that this process ultimately makes a fundamental contribution to reading efficiency (Rastle, 2019).

Research on the relationship between reading skills and print exposure has demonstrated that reading ability is a strong predictor of how much children choose to read (e.g., Leppänen et al., 2005; van Bergen et al., 2018). The CYP-LEX data demonstrate why a failure to acquire good phonic and morphological knowledge early in the reading acquisition is likely to have a negative snowball effect on a child’s reading habits. Because the number of rare and morphologically complex words increases as children transition through primary and into secondary school, texts will often be too difficult for readers with suboptimal reading skills, which is likely to result in an unrewarding reading experience and, consequently, a desire to expose themselves to even less text (cf. Allington, 1984).

The CYP-LEX findings also demonstrate why reading widely, both during primary and secondary education, is pivotal if one is to become a skilled reader—only by reading widely can a child see as many words as possible as frequently as possible. We have said above that it is crucial that children discover the underlying morphological regularities between spelling and meaning early on. In English, prefixes and suffixes typically do not occur on their own (e.g., Grainger & Beyersmann, 2017; Schreuder & Baayen, 2017), and, therefore, to discover these minimal meaning-bearing units and to elucidate their meanings, a reader needs to rely on experience with whole words and in many different contexts (e.g., Tamminen et al., 2015). Because the vast majority of words in children’s books are only encountered a few times and in a small subset of books, only those children who read widely will be able to experience different monomorphemic and multimorphemic words frequently and in a wide range of contexts and, hence, develop high-quality lexical representations. Thus, our findings suggest that children need strong decoding skills and sound morphological knowledge in order to be able to read, understand what they are reading, and derive pleasure from it, but that improvement and fine-tuning of these skills is only possible if children read widely.

To conclude, this article has introduced CYP-LEX, a new large-scale publicly available lexical database of books popular with British children and young people. CYP-LEX provides a wide range of lexical statistics derived from book vocabulary in three age bands (7–9, 10–12, 13+), thus enabling researchers studying reading to conduct different linguistic and psycholinguistic investigations. Initial analyses of the CYP-LEX data suggest that children may encounter many words in books that they have not previously encountered on British television, and that each age band contains many words that do not appear in books for younger children. Moreover, the absolute majority of words in each age band are low in frequency, and books within and across the age bands differ substantially regarding which of these low-frequency words they make use of. These initial findings suggest that orthographic learning may occur only for a small proportion of the words to which children are exposed when they read. Therefore, in order to become skilled readers, child-ren need to develop strong phonic and morphological analy-sis skills as early as possible, while they also need to read widely throughout both primary and secondary education.

## Notes

1. In this article, we do not discuss picture books as they target younger (pre-school) children (0–5 years) and are typically not used for independent reading (but see Dawson et al., 2021; Green et al., 2023; Massaro, 2015; Montag et al., 2015, for analyses of picture book corpora of different sizes). We also do not discuss lexical databases available for languages other than English, but we refer the reader to Soares et al., 2014, for Portuguese, Lété et al., 2004, for French, Schroeder et al., 2015, for German, Terzopoulos et al., 2017, for Greek, and Li et al., 2023, for Chinese.

2. For earlier lexicographic work, see Thorndike (1921), who compiled a 10,000-word list (“The Teacher’s Word Book”) from texts such as the Bible, English classics, and textbooks that children were supposedly reading at the time; Knowles and Malmkjær (1996), who developed a 760,000-word corpus of children’s books written in the 19th–20th centuries and another corpus (of similar size) of contemporary children’s fiction; and Zeno et al. (1995), who developed word frequency lists for American English. Neither of the first two databases is publicly accessible.

3. For information on accuracy and reliability of the Stanford CoreNLP software, we refer the reader to its official webpage (https://stanfordnlp.github.io/CoreNLP/) and the Stanford NLP group’s publications page (https://nlp.stanford.edu/pubs/).

4. Like in any other lexical database, these (and all following) numbers are approximations (e.g., Sampson, 2002) and ne-cessarily depend on the algorithms implemented in the software used to process the raw text files. Given the size of the corpus, it is not possible to provide error rates or manually correct the output of these algorithms. Nonetheless, a careful application of the state-of-the-art methods in this article allows us to be confident that provided statistics are valid and reliable.

5. Because of how the Zipf frequency is calculated, the lowest Zipf frequency for a given corpus necessarily depends on the corpus size. For instance, in a corpus that has 1 million words, the lowest possible Zipf frequency for words not observed in the corpus is 3 (1 occurrence per million words); however, if a corpus contains 10 million words, the lowest possible Zipf frequency for words not attested in the corpus is 2. In most cases, this should not have any important consequences. Nevertheless, if one wishes to compute a word’s Zipf frequency across the entire CYP-LEX corpus, it is not advisable to average across this word’s Zipf frequencies in the individual age bands. Instead, a better approach is to sum up the raw counts across the age bands and then apply the Zipf transformation. That way, the resulting value will be less influenced by the fact that the age bands have different sizes.

6. We acknowledge that whether a certain word can and should be considered a function word can be contentious, and we therefore used the list of function words provided in the NLTK Python library. Our aim was to have a list that was as short as possible but contained words which would be classified as function words by most researchers and algorithms. The resulting list contained 185 word forms and can be found in Supplementary Material C (https://doi.org/10.17605/OSF.IO/SQU49).

7. In this case, the randomly selected book was “Code Name Bananas” by David Walliams.
